# Multisite Assessment of Methods for Cell Preservation Upstream of Single-Cell RNA Sequencing

**DOI:** 10.7171/001c.162768

**Published:** 2026-06-08

**Authors:** Fred W. Kolling IV, Jessica W. Podnar, Owen Wilkins, Claire J. Fraser, Madolyn L. MacDonald, Shawn W. Polson, Zachary T. Hebert, Sridar V. Chittur, Andrew Hayden, Marcy Kuentzel, Michael Heinz, Gabriella M. Huerta, Holly S. Stevenson, Aditi Karmakar, Catrina Fronick, Lisa Cook, Sean Vargas, Xiaoling Xuei, Patrick McGuire, Molly Zeller, Yanping Zhang, Ru Dai, Xinkun Wang, Ching Man Wai, Jyothi Thimmapuram, Devender Arora, Tania Mesa, Jun Fan, Yuriy O. Alekseyev, Francis Cervone, Christopher Williams, Nickolas Gorham, Alexander Lemenze, Sara Goodwin, Jonathan Preall, Charles A. Whittaker

**Affiliations:** 1 Dartmouth Cancer Center Dartmouth College; 2 Genomic Sequence Analysis Facility University of Texas Austin; 3 Flow Cytometry and Genomics Core Facility Barrow Neurological Institute; 4 Bioinformatics Data Science Core University of Delaware; 5 Bioinformatics Data Science Score University of Delaware; 6 Molecular Biology Core Facilities Dana-Farber Cancer Institute; 7 Center for Functional Genomics and Department of Biomedical Sciences University at Albany; 8 McDonnell Genome Institute Washington University School of Medicine; 9 Genomic Sequencing and Analysis Facility University of Texas; 10 Genomics Core University of Texas at San Antonio; 11 Center for Medical Genomics Indiana University School of Medicine; 12 Biotechnology Center University of Wisconsin Madison; 13 ICBR Gene Expression and Genotyping University of Florida; 14 NUSeq Core, Center for Genetic Medicine Northwestern University Feinberg School of Medicine; 15 Bioinformatics Core Purdue University; 16 Molecular Genomics Core Moffitt Cancer Center; 17 Molecular Genomics Core Texas A&M Institute for Genomics Sciences and Society; 18 Single Cell Sequencing Core and Microarray and Sequencing Resource Core Boston University; 19 Molecular and Genomics Informatics Core Rutgers New Jersey Medical School; 20 Cold Spring Harbor Laboratory Cold Spring Harbor Laboratory; 21 Barbara K. Ostrom (1978) Bioinformatics and Computing Core Facility, Koch Institute Massachusetts Institute of Technology

**Keywords:** single cell genomics, 10x Genomics, Parse Bioscience, Honeycomb Bio, Illumina, DNA Sequencing Research Group, Cell Preservation

## Abstract

**Introduction/Objective:**

Single-cell RNA sequencing (scRNA-seq) resolves cell types and molecular phenotypes within heterogeneous specimens but typically requires fresh, high-quality single-cell suspensions processed immediately to preserve transcriptional profiles. This constraint complicates samples with long preparation times and prevents collection at remote sites lacking single-cell instrumentation. Several commercial assays now enable preservation at the point of collection through fixation or cryopreservation, allowing processing to occur months later. The Association of Biomolecular Research Facilities’ DNA Sequencing and Genomics and Bioinformatics Research Groups undertook a cross-platform, multisite study to assess the performance and reproducibility of three such platforms: 10x Genomics FLEX, Parse Biosciences Evercode WT v2, and Honeycomb Bio HIVE.

**Materials and Methods:**

Total leukocytes and peripheral blood mononuclear cells (PBMCs) were isolated from a single healthy individual, with EasySep reagent used for red blood cell depletion of the leukocyte fraction. Cells were characterized by a 21-color flow cytometry panel to provide a reference, and the remaining material was fixed or cryopreserved according to each platform’s protocol. Preserved leukocyte samples were prepared in parallel by two technicians (“A” and “B” replicates) and distributed to multiple ABRF member core facilities for downstream processing, while fresh leukocytes processed with the 10x 3’ v3.1 (3pGEX) chemistry served as a reference. Libraries were sequenced at a central site, and performance was evaluated across standard scRNA-seq quality control metrics, gene and transcript detection sensitivity, cell-type discovery and annotation, differential expression, and correlation analyses.

**Results:**

Data from all platforms integrated effectively and produced concordant results for cell-type annotation and relative abundance, with cell-type proportions broadly consistent with the flow cytometry reference. However, platform-specific expression signatures were evident for a subset of genes, and cross-site reproducibility varied between methods, with the FLEX workflow showing greater susceptibility to technical variation introduced during on-site sample processing. Preservation-based methods (FLEX and HIVE) showed better retention of fragile granulocyte populations than fresh samples processed with the 10x 3pGEX workflow.

**Discussion:**

Improvements to preservation methods are changing how single-cell research is conducted by decoupling sample collection from downstream processing. Our investigation into the performance and reproducibility of each platform provides a resource to help investigators and core facilities select the most appropriate single-cell preservation workflow given their sample type, cell populations of interest, sample collection logistics, and laboratory infrastructure constraints.

## Introduction

In recent years, single-cell RNA sequencing (scRNA-seq) has emerged as a powerful tool to measure gene expression within individual cells, providing the resolution required to determine cell identity and define cell states. These methods rely on microfluidically generated droplets,^1^ picowells,^2^ or the cells themselves^3^ to partition and uniquely barcode the RNA molecules within each cell.^3,4^ The barcoded RNAs are then sequenced to determine their identity and the cell of origin. The starting material for these experiments is a suspension of single cells that was produced either through the dissociation of tissues or purification of cells from biofluids (e.g., blood). The integrity of the cells in suspension is critical and has a direct impact on the quality of data from scRNA-seq experiments. Cells that are stressed or undergoing apoptosis generate distinct gene expression signatures and actively degrade their mRNAs, thus reducing transcriptome complexity.^5–7^ Furthermore, cell lysis causes the release of RNAs into the cell suspension, and these “ambient” RNAs can become barcoded and erroneously associated with cell expression profiles, which decrease the cell-specific signal.^8,9^ For these reasons, single-cell experiments have required optimized dissociation procedures using fresh, high-viability single-cell suspensions for use in these assays. This presents a challenge when large numbers of samples are processed in parallel, or when the reagents and instrumentation for single-cell capture are distant from where the cell suspension is generated. To circumvent these issues, methods such as methanol fixation and cryopreservation have been validated in specific sample types (e.g., blood, cerebrospinal fluid, etc.)^3,10–14^; however, their impact on cell type representation and transcript detection in some contexts has prevented their widespread use.^15^

Recently, several commercial solutions including 10x Genomics FLEX, Parse Bioscience Evercode, and Honeycomb Bio HIVE have come to market to enable preservation of single-cell samples upstream of scRNA-seq. Separating the preparation of single-cell suspensions (from cell capture, library preparation, and sequencing) makes it possible to collect samples in one location and ship to another for processing. As a result, core facilities and other service providers will be eager to implement these methods to simplify sample collection logistics for internal users and to facilitate access by external clients to single-cell services. However, there are many variables to consider when choosing a platform including sample preservation (fixation or cryopreservation) and RNA capture (Poly-dT priming, random priming, or probe-based detection) methods between platforms, as well as the instrumentation and hands-on time required. Differences in cell capture and sequencing performance have been described in comparative studies evaluating various scRNA-seq methods.^16–19^ This study provides additional insight into the impacts of prolonged storage time and kit reproducibility across multiple performance sites, as well as considerations when selecting a given chemistry.

To evaluate the performance, reproducibility, and workflow requirements of the available methods, the Association for Biomolecular Research Facilities’ (ABRF) DNA Sequencing and Genomics and Bioinformatics Research Groups have conducted a multisite, cross-platform assessment of the 10x Genomics FLEX, Honeycomb HIVE, and Parse Evercode technologies. We show that while there is general agreement in cell-type assignment, relative abundance, and overall gene expression profiles across platforms and performance sites, strong technology-specific expression signatures exist within the data and cross-site reproducibility varies between methods. In addition, we find that sample preservation by fixation (FLEX) and freezing (HIVE) improves the retention of fragile granulocyte populations compared to fresh sample processing using the traditional 10x 3’ GEX (3pGEX) assay. This study demonstrates the importance of selecting an appropriate single-cell technology based on experimental and logistical constraints and also provides a resource to core facilities and individual users to make informed decisions when implementing these assays.

## Methods

### Study Design

The study was designed to evaluate reproducibility, ease of use, and performance of three technologies for cell preservation on the same sample type across three commercially available options. We selected a single site to collect and preserve the samples and nine ABRF member genomics core facilities to perform downstream processing. To assess variability in sample preservation, preservation protocols were performed by two different technicians in parallel to produce “A” and “B” replicates. Once preservation protocols were completed, the samples were stored at -80°C for two weeks before shipping to each of the participating sites. Prior to any site receiving their samples, they were provided with hands-on training by the respective vendors using control samples. After two weeks, each site received “A” and “B” preserved samples that were stored for an additional two weeks at -80°C for a total of four weeks of storage time before completing the assigned workflow. Sequencing ready libraries were shipped to a central site for quality assessment and sequencing. Our follow-on study using peripheral blood mononuclear cells (PBMCs) was performed at a single site following the same protocols described for the total leukocyte experiment except in the cases noted in the following section. It should be noted that comparisons across platforms are not strictly symmetrical: the 10x FLEX workflow was compared to the fresh 10x 3’ GEX (3pGEX) control, whereas the Honeycomb HIVE comparison was between cryopreserved samples stored for 1 day versus 28 days. This reflects a deliberate design choice: the HIVE technology is a cryopreservation-based method and does not have a “fresh” processing mode equivalent to the 3pGEX assay. The 1-day HIVE samples therefore serve as the earliest practical reference point for that platform, and the 3pGEX fresh sample serves as the overall fresh reference across all comparisons.

### Sample Collection, Cell Isolation and Quality Control (QC)

Blood collection from a single healthy male was performed at Dartmouth Hitchcock Medical Center under an institutional review board approved protocol. Approximately 50 mL of whole blood was collected into Ethylenediaminetetraacetic acid (EDTA) vacutainer tubes (BD Bioscience, Franklin Lakes, NJ, USA) and immediately transported to the Immune Monitoring Lab at Dartmouth Cancer Center for processing. For total leukocyte isolation, the blood sample was subjected to two rounds of RBC depletion using magnetic beads from the EasySep RBC Depletion kit (StemCell Technologies, Vancouver, Canada) and generating about 8 x 10^^7^ cells. Two additional washes were performed with samples spun at 500 x g for 10 minutes to reduce the presence of platelets. For PBMC isolation, gradient separation of the blood was performed using Histopaque-1077 (Corning Life Sciences, Durham, NC, USA). Briefly, blood was overlaid onto the Histopaque-1077 and then centrifuged for 30 minutes at 750 x g. An additional spin was performed with samples at 500 x g for 10 minutes to reduce the presence of platelets. For both sample types, cells were transferred to the Dartmouth Genomics Shared Resource for counting and viability assessment on a Nexcellom K2 instrument (Revvity, Waltham, MA, USA) prior to running each of the workflows as described in the following section. Both total leukocyte and PBMC samples exhibited >95% viability as determined by acridine orange/propidium iodide (AO/PI) staining.

### Flow Cytometry

Cells were stained for flow cytometry analysis as follows. 2x10^6 total leukocytes or PBMCs were suspended in 1.25 µg/mL of human immunoglobulin G (IgG) to block Fc receptors and incubated for 10 minutes in a 5 mL flow cytometry assisted cell sorting (FACS) tube. Following this, 5 µL of Brilliant Stain Buffer Plus (BD Biosciences) and 5 µL of True-Stain Monocyte Blocker (BioLegend, San Diego, CA, USA) were added in succession. Due to steric hindrance preventing its use in a master mix of antibodies, anti-TCRγδ was added, and cells were incubated for 10 minutes at room temperature (RT) in the dark. The remaining antibodies were then added in a master mix, and the cells were incubated for 30 minutes at RT in the dark. All antibodies used are listed in Supplemental Table 1. To wash unbound antibodies, 3 mL of phosphate-buffered saline were added and cells were centrifuged at 500 x g for 5 minutes. Cells were resuspended in 1% paraformaldehyde in phosphate-buffered saline to fix the antibodies to the cells and then washed as previously noted. Cells were then acquired on a 5-laser Aurora Spectral Cytometer (Cytek Biosciences, Fremont, CA, USA).

### 10x Genomics 3’ v3.1 on Fresh Specimens

Following QC, cells were loaded onto two separate lanes of a single-cell Chip G to generate “A” and “B” replicates and processed on a 10x Chromium X instrument, which targets 10,000 cells per sample. This A/B replicate design applied to the total leukocyte sample only; the PBMC follow-on experiment was processed as a single library using the same 3’ v3.1 workflow. Emulsions containing encapsulated single cells were removed from the chip and processed according to the Chromium Next Gel Beads-in-Emulsion (GEM) Single-cell 3’ v3.1 User Guide (CG000204 RevD). Completed libraries were examined on a Bioanalyzer (Agilent Technologies, Santa Clara, CA, USA) and quantified by Qubit (Thermo Fisher Scientific, Waltham, MA, USA) prior to pooling and loading on a NovaSeq6000 instrument as described in the following section.

### Sample Preservation

Following collection, cell isolation, and QC, samples were processed with their respective preservation protocols: cell fixation or cryopreservation followed by storage at -80°C prior to scRNA-seq library generation. To ensure the highest cell quality and minimize sample variation for all methods, a plan was formulated to process the samples for preservation in parallel at the central site followed by distribution to the testing sites. Details can be found in the Cell Preservation Supplemental Protocol.^20^ Cells for the 10x FLEX RNA profiling kit followed the Fixation of Cells & Nuclei for Chromium Fixed RNA Profiling demonstrated protocol (CG000478, Rev A) using 1 million cells per fixation. Cells for the Parse workflow were fixed using the Evercode Cell Fixation v2 kit (ECF2001) following the Evercode Fixation User Manual V2.0.1 with 3 million cells per fixation. Honeycomb HIVEs were loaded with 15,000 cells into v1 HIVEs following the HIVE scRNA-seq v1 sample capture user protocol (Rev A) for the leukocyte samples, and 30,000 into the CLX HIVEs for PBMCs following HIVE CLX scRNA-Seq Sample Capture protocol.

### 10x Genomics FLEX Sample Processing on Fixed Cells

10x FLEX single-cell libraries were completed with the Chromium Fixed RNA Profiling kit (part number 1000474) to process fixed cells following user guide CG000477 Rev A. Cell fixations were completed by a central site, and aliquots with 1 million fixed cells were shipped on dry ice to each of the testing sites. Briefly, fixed and permeabilized cells underwent probe hybridization and ligation for 16 hours, partition of GEM and barcoding, cDNA amplification, and library construction each with unique indices following the protocol of 10x Chromium Fixed RNA Profiling Reagent Kits User Guide, CG000477 RevA (10x Genomics, Pleasanton, CA, USA). For the leukocytes, each testing site processed replicate “A” and “B” to produce a total of eight libraries, and a single library was generated for the PBMC sample. The targeted recovery per sample was 10,000 cells per library. The quality and quantity of each library was checked with the Qubit (Thermo Fisher Scientific), Agilent Bioanalyzer 2100 HS DNA kit (Agilent Biotechniques), and qPCR using the Kapa SYBR Fast RT-qPCR kit, KK4602 (Roche Diagnostics, Basel, Switzerland).

### Honeycomb HIVE Sample Processing

Honeycomb single-cell libraries were completed following the HIVE scRNA-seq V1 or CLX Processing kit user protocols (Honeycomb Biotechnologies, Inc.). V1 kit Rev A was used for leukocytes and CLX for the extension study with the PBMCs. The central site that loaded the HIVEs used the HIVE scRNA-seq complete kit (HCB018) that included reagents and materials for sample loading and processing while the testing sites used the HIVE scRNA-seq Starter Bundle (HCB019) for sample processing only. HIVEs were shipped from the central site to testing sites on dry ice for processing. Briefly, on day 1, the cell loaded HIVE collectors were thawed and washed followed by cell lysis and a hybridization step to capture Poly-A transcripts from individual cells in the HIVE picowells on the beads. The beads were recovered following the HIVE protocol and then transferred to a 96 well filter plate to perform first and second strand synthesis. Steps were completed in the 96 well filter plate utilizing vacuum assembly provided by the manufacturer as part of the HIVE scRNA-seq Starter Bundle. Following the second strand synthesis, a whole transcriptome amplification step was performed. Protocol was continued on day 2 to complete the library prep, SPRI clean-up of whole transcriptome amplification product was followed by an additional polymerase chain reaction to incorporate the Illumina sequencing adapters and indices using a total of 25 ng per reaction. For the leukocytes, each testing site processed replicate “A” and “B” to produce a total of eight libraries, and a single library was generated for the PBMC sample. The quality and quantity of each library was checked with the Qubit (Thermo Fisher Scientific), Agilent Bioanalyzer 2100 HS DNA (Agilent Biotechniques), and RT-qPCR using the Kapa SYBR Fast qPCR Kit, KK4602 (Roche Diagnostics).

### Parse Biosciences Sample Processing

The Parse single-cell library was prepared with the Parse Single-Cell Whole Transcriptome Kit, Evercode WT Mini v2, ECW02010 (Parse Biosciences) following the user manual V2.0.1. For the first round of barcoding, 4000 cells were distributed into each of the 12 wells (48,000 cells total) with subsequent pooling and barcoding steps performed following the user guide. Two sublibraries were completed for the sample and a single sublibrary was sequenced. The quality and quantity of the sequencing library was checked with the Qubit (ThermoFisher Scientific) and Agilent Bioanalyzer 2100 HS DNA Kit (Agilent Biotechniques).

### Illumina Sequencing

Sequencing was completed at one site using the NovaSeq 6000 instrument from Illumina. Libraries generated from the leukocytes for 10x were sequenced on a S2 100 cycle flow cell 28-10-10-90 targeting 10,000 reads per cell for FLEX and 25,000 reads per cell for the 3’ GEX libraries. Pooled loading concentration for the 10,000 libraries standard workflow was 1500 pM. The Honeycomb leukocyte libraries were sequenced on a full S1 100 cycle flow cell 25-8-8-5 targeting 25,000 reads per cell. The pooled loading concentration for Honeycomb libraries standard workflow was 1800 pM. 10x and Parse PBMC libraries were sequenced on individual lanes using the XP workflow with a SP 200 cycle flow cell 100-10-10-100; pooled loading concentration was 750 pM, and the Honeycomb PBMC library was sequenced on a full SP 100 cycle flow cell, 25-8-8-50. Prior to sequencing, qPCR was completed to determine library concentration for pooling prior to loading the sequencer. The Honeycomb libraries required custom primers, which are provided in the sample processing kit with the library prep reagents at 100 uM. The NovaSeq 6000 Custom Primer protocol, Document 1000000022266 v03, was followed to prepare and load the custom primers for the Honeycomb sequencing runs.

### Data Analysis

Sequencing data (FASTQ format) was processed to generate cell-level feature barcode matrixes using vendor software. For leukocyte samples, the 10x samples were processed using Cell Ranger v7.1.0, and data from all valid barcodes (raw_feature_bc_matrix data) was used in downstream analysis. Honeycomb leukocyte data was processed using BeeNet v1.1.3 specifying 10,000 barcodes. The 10x PBMC samples were processed using Cell Ranger v7.1.0 and only cell-associated barcodes were used for downstream analysis (filtered_feature_bc_matrix data). The PBMC Honeycomb sample was processed using BeeNet v1.1.3, which specifies 15,000 barcodes. The PBMC Parse Evercode data was processed using split-pipe v1.0.5p with default settings.

Following data generation, processing and analysis were carried out in a contained R environment that includes a variety of data science, genomics, and single-cell RNA-Seq software packages (https://github.com/GBIRG/scRNAseq_2022/tree/main/Docker, Fig. S1). The contained computer solution we have implemented facilitates distributed and collaborative data analysis projects, and the image is made available to facilitate public analysis of this dataset (docker://alemenze/abrfseurat). A listing of all the packages and versions is available in the supplementary file DSRG_GBIRG_Rcontainer_sessionInfo.txt. In order to capture low-information content granulocytes in the leukocyte samples, data were imported into “Seurat” version 4.3.0^21^ using the following low-stringency parameters: nFeature_RNA > 30, nCount_RNA > 100, mitoRatio > 0.1, and genes with nonzero counts in at least 10 cells. The data from each platform were then normalized using *sctransform*^21^ and integrated using anchor-based integration^22^ followed by dimensionality reduction and clustering.

### Cell-Type Assignment

Clusters were then characterized with an integrated assessment of cluster-specific marker genes, transcriptional complexity, and scoring of expression signatures with ‘singleR.’^23^ In order to assign cell types to each leukocyte (Figure 3A) and PBMC (Figure 3C) cluster, ‘singleR’ was used with the ‘celldex’^24^ references: Human Primary Cell Atlas,^25^ Database Immune Cell Expression,^26^ and Blueprint Encode.^27^ ‘SingleR’ assigns identity at the cellular level. To assign cell types to unsupervised clusters, the fraction of cells assigned to each identity was calculated for each cluster and assignments that were coherent on a cluster-level were prioritized. In addition to ‘singleR,’ cluster-specific gene markers were identified using *Find All Markers*, and these marker lists were examined using the MsigDB Investigate Gene Sets^28,29^ utility and the Cell Type Signature (C8) collection. Cell-type assignment conclusions were then manually validated by examining cell-type specific markers. Debris (cell-free or empty droplets) clusters were defined as those dominated by cells or droplets with poor QC metrics, ambiguous cell-type assignments using ‘singleR’, and no cluster-specific markers. Clusters meeting these criteria were flagged as debris and excluded from downstream analyses (Figure S2).

**Figure:**
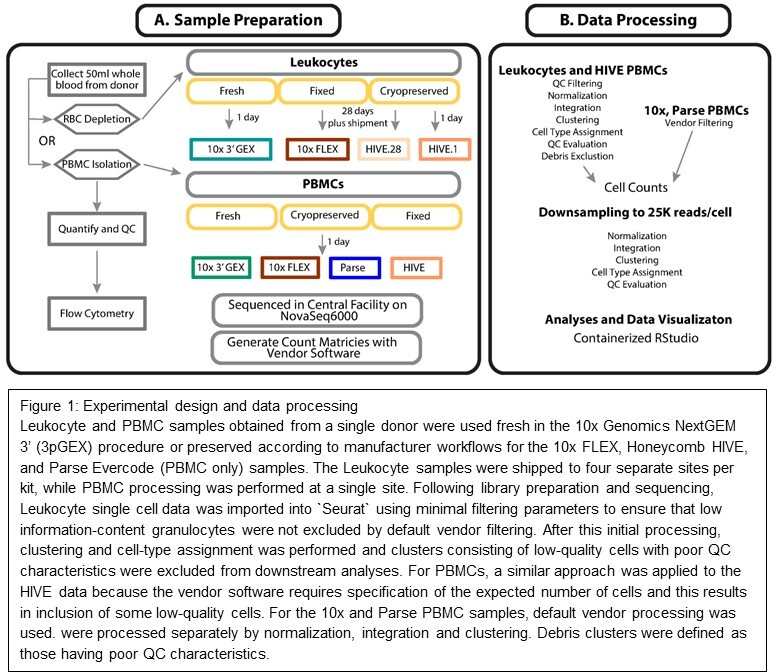


**Figure:**
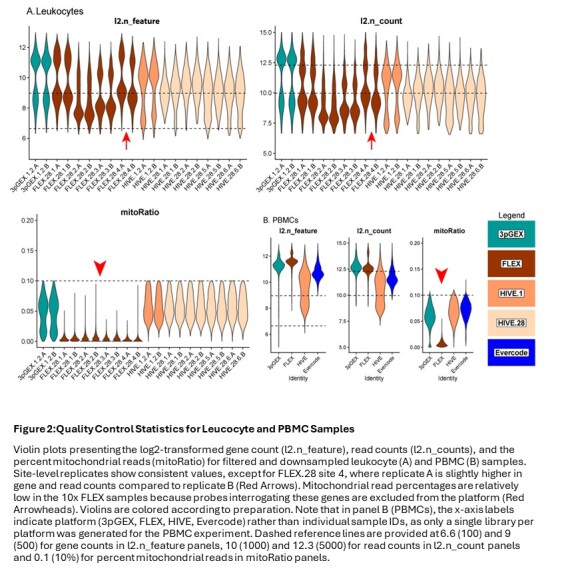


**Figure:**
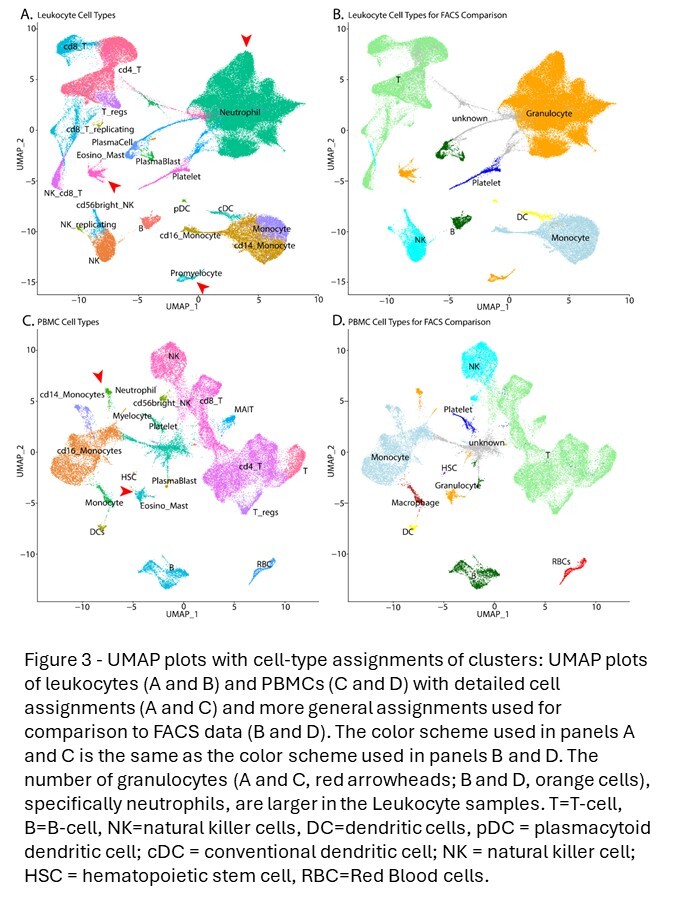


To remove potential sequencing depth-related biases in downstream analysis, the sample-level cell counts following debris exclusion were used as denominators to downsample the raw data to a sequencing depth of 25,000 reads per cell. The resulting FASTQ files were then used as input to the same analysis procedure. For the PBMC data, vendor software was used to exclude debris clusters from the 10x 3pGEX, FLEX, and Parse samples. For the HIVE samples, 15,000 cells were requested in processing and a similar cluster-level debris exclusion process as used in the leukocytes was applied. As in the case of the leukocytes, once a reliable cell count was obtained, FASTQ files were downsampled to 25,000 reads per cell and analysis was repeated. Cell-type assignments prepared for the predownsampling object were then transferred to the downsampled object so that cells flagged as low-confidence coils could be excluded from the analysis of downsampled data. These metadata were then reevaluated in the context of the downsampled data to revise and finalize cell-type assignments.

For comparison with transcript coverage over gene bodies, the Python package deepTools v3.5.4^31^ was used to generate bigWig coverage files from the binary alignment map (BAM)-formatted alignments for each sequencing platform. Normalized coverage over exons was computed using deepTools computeMatrix with the following parameters: scale-regions -b 100 -a 100, unscaled5prime 60, unscaled3prime 60, skipZeros, and metagene and gene annotations, which are gene transfer format (GTF) files provided by 10x Genomics (GRCh38-2020-A, based on GENCODE v32/Ensembl 98).

### Correlation Analysis

To agnostically evaluate the similarity between samples across technologies and sites, we applied a bulk correlation analysis across the dataset. SCT normalized expression counts were aggregated per sample using the “Seurat” function AggregateExpression on single-cell transform (SCT) assay data and Pearson correlations were calculated between each pairwise sample for downstream visualization.

### Differential Expression Analyses

To perform a multisite, cross-platform assessment, we executed comparisons between A and B replicates generated at each site and also between A replicates from each site and 28-day and 1-day storage time. To ensure consistent comparisons across analyses, we filtered to show only genes present in the FLEX probeset, which includes 28,690 genes to make the comparisons uniform across each platform. For each comparison, count data for relevant replicates were extracted from the object, and differential expression analysis using ‘DESeq2’^30^ and an approximate condition design was then performed. Differentially expressed genes (DEGs) were defined as genes having an absolute log2 fold change (log2FC) of >1 and a false discovery rate (FDR) adjusted p-value (padj) of <0.05. The results we visualized using enhanced volcano plots were generated for each comparison using ‘EnhancedVolcano,’^31^ and fold change box plots showing A_vs_B and site-specific variation were generated using ‘ggplot2.’^32^

### Unique Molecular Identifier (UMI) Gene Body Coverage Analysis

For sequencing saturation analyses, BAM files were downsampled to a range of different depths relative to the starting library using PySAM v0.18.0. Reads were then recounted for unique UMI, gene, and barcode sets. In brief, only reads originally tagged by Cell Ranger with the ‘xf’ tag equal to 17 (read mapped confidently to the transcriptome and to one unique feature) or 25 (read mapped confidently to the transcriptome and to one unique feature with a read that is representative for a molecule and is treated as a UMI count) were added to sets for each gene and barcode pair, resulting in a matrix that faithfully reproduces the Cell Ranger counting rubric at each depth. To extrapolate the number of predicted mean UMIs/cell at saturation, downsampled data was fit to a Michaelis-Menten model of the following form:

$U = $

Where:

$U$ = mean UMIs per cell

$U_{max}$ = UMIs per cell at saturation

$K_{r}$ = 50% saturation point (in mean reads per cell)

$R$ = mean reads per cell

## Results

We compared data generated by three different single-cell RNA-Seq technologies (10x FLEX, Honeycomb HIVE, and Parse Evercode) that enable users to preserve cells upstream of scRNA-seq processing prior to single-cell capture and library preparation. Total human leukocytes (PBMCs and granulocytes) were profiled due to the well-established cell-type-specific expression profiles of PBMCs and the potential for preservation-focused scRNA-seq protocols to capture granulocytes, which have been underrepresented in single-cell data to date due to their fragility and low transcriptional complexity. To establish a reference to many existing datasets, fresh leukocytes were processed using the 10x Genomics 3’ v3.1 chemistry (3pGEX). To minimize the number of variables in our study, the total leukocyte and PBMC samples were collected from the same individual at a single site. Fixation and storage were performed in parallel by two technicians (designated “A” and “B” replicate samples) to assess technical variability in sample preparation. Preserved samples from the FLEX and HIVE were shipped to four separate sites and processed after 28 days in storage to assess the reproducibility of these methods and identify possible sources of cross-site variation. The A/B replicate design and multisite distribution described here applied exclusively to the total leukocyte phase of the study; the PBMC follow-on experiment was performed at a single site and processed after one day of storage as described in the following section. Kit compatibility challenges with total leukocytes in the Parse Evercode chemistry prevented their inclusion in the initial phase of this study, so a second phase was conducted to generate data from all three platforms. This follow-on experiment was performed on PBMC specimens isolated at the same site as the first phase and processed using the 10x 3pGEX (fresh), 10x FLEX, HIVE, and Evercode workflows. Table 1 presents the experimental metadata associated with the 20 leukocyte samples and 4 PBMC samples. Each sample was sequenced at a central location to the manufacturer recommended depth (Table 1, ReadCount, Figure S3A). Count data were prepared using vendor-specific tools, and the resulting data were imported into “Seurat” v4^21^ for analysis.

**Table 1.** Experimental metadata featuring a total of 24 samples that were considered in this experiment (20 leukocyte samples and 4 PBMC samples). The nomenclature presented in the sample column is used in sample-level visualizations. The color key indicates experimental groups in subsequent figures.

**Sample**	**Cells**	**ID**	**company**	**prep**	**version**	**day**	**site**	**replicate**	**Filtering**	Read Count*	Cells**	**Color Code**
3pGEX.1.2.A	Leukocyte	10x-23	10x	3pGEX	3.1	1	2	A	Custom	740023039	6880	
3pGEX.1.2.B	Leukocyte	10x-24	10x	3pGEX	3.1	1	2	B	Custom	788767980	7210	
FLEX.28.1.A	Leukocyte	10x-21	10x	FLEX	1	28	1	A	Custom	375927550	10764	
FLEX.28.1.B	Leukocyte	10x-22	10x	FLEX	1	28	1	B	Custom	344076798	12650	
FLEX.28.2.A	Leukocyte	10x-25	10x	FLEX	1	28	2	A	Custom	315635895	8706	
FLEX.28.2.B	Leukocyte	10x-26	10x	FLEX	1	28	2	B	Custom	366609531	9736	
FLEX.28.3.A	Leukocyte	10x-27	10x	FLEX	1	28	3	A	Custom	411703884	8596	
FLEX.28.3.B	Leukocyte	10x-28	10x	FLEX	1	28	3	B	Custom	300570611	9255	
FLEX.28.4.A	Leukocyte	10x-29	10x	FLEX	1	28	4	A	Custom	391058232	9799	
FLEX.28.4.B	Leukocyte	10x-30	10x	FLEX	1	28	4	B	Custom	398164770	10123	
HIVE.28.1.A	Leukocyte	HC-11	HC	HIVE	v1	28	1	A	Custom	168784657	4684	
HIVE.28.1.B	Leukocyte	HC-12	HC	HIVE	v1	28	1	B	Custom	192953268	2953	
HIVE.1.2.A	Leukocyte	HC-13	HC	HIVE	v1	1	2	A	Custom	233183826	3279	
HIVE.1.2.B	Leukocyte	HC-14	HC	HIVE	v1	1	2	B	Custom	230076516	3706	
HIVE.28.2.A	Leukocyte	HC-15	HC	HIVE	v1	28	2	A	Custom	160258808	4012	
HIVE.28.2.B	Leukocyte	HC-16	HC	HIVE	v1	28	2	B	Custom	164240099	5165	
HIVE.28.5.A	Leukocyte	HC-17	HC	HIVE	v1	28	5	A	Custom	162500315	3882	
HIVE.28.5.B	Leukocyte	HC-18	HC	HIVE	v1	28	5	B	Custom	181172979	5140	
HIVE.28.6.A	Leukocyte	HC-19	HC	HIVE	v1	28	6	A	Custom	151699896	2934	
HIVE.28.6.B	Leukocyte	HC-20	HC	HIVE	v1	28	6	B	Custom	179175285	2297	
pbmc.10x3p	PBMC	pbmc.10x3p	10x	3pGEX	3.1	1	2	A	Vendor	448023652	9791	
pbmc.10xFlex	PBMC	pbmc.10xFlex	10x	FLEX	1	1	2	A	Vendor	585993045	2553	
pbmc.HC.v2	PBMC	pbmc.HC.v2	HC	HIVE	v2	1	2	A	Custom	434804102	11826	
pbmc.Parse	PBMC	pbmc.Parse	Parse	Evercode	v2	1	2	A	Vendor	998478660	10409	

* Total reads before downsampling

** Cell count used as denominator in downsampling

Except for the 10x and Parse PBMC samples, the initial processing used permissive filters for transcriptional complexity to ensure retention of granulocytes. These unfiltered data were then integrated and clustered, and cluster-specific markers were identified by differential expression analysis. Cell-type assignment was performed using ‘singleR’ and by examination of cluster-specific marker genes. Clusters with poor QC parameters, ambiguous cell types, and no clear distinguishing marker genes were flagged as cell-free droplets and excluded from downstream analysis (Figure S2). To standardize the per-cell sequencing depth for different samples (Figure S3C), the counts of high-confidence cells (Table 1, Cells; Figure S3B) were then used as a denominator to downsample the original FASTQ files to a consistent sequencing depth of 25,000 reads per cell (Figure S3D).

Following downsampling of the FASTQ files and reprocessing using vendor software, data were subset to droplets labeled as cells in the initial analysis of the full dataset. Traditional QC metrics, including number of genes per cell (n_feature), number of UMIs per cell (n_count), and the fraction of reads derived from the mitochondrial genome (mitoRatio), were evaluated to compare each platform. For the leukocyte data (Figure 2A), the site-level A and B replicates are highly consistent except for those from site 4 (FLEX.28.4A and FLEX.28.4B, red arrows). In addition to this FLEX replicate variability for site 4, there is variability in both the number of genes and the number of counts within the FLEX sets. Samples from sites 1 and 4 (FLEX.28.1.A/B and FLEX.28.4.A/B) have more genes and counts than samples from sites 2 and 3 (FLEX.28.2.A/B and FLEX.28.3.A/B). The number of genes and counts observed in the site 1 and 4 FLEX samples is similar to what was observed in the 10x 3pGEX control. Transcriptional complexity was consistent across all four HIVE 28-day site samples; however, higher counts were observed in 1-day samples compared to those after 28 days of storage, and this difference may be pronounced in monocytes, B-cells, and Dendritic Cells (DCs). During import, cells with greater than 10% mitochondrial reads were excluded from consideration, which is reflected in the plots of this parameter. Notably, the 10x FLEX technology lacks probes targeting mitochondrial ribosomal protein genes, which account for most mitochondrial transcripts. This results in a lower proportion of reads attributed to mitochondrial expression (red arrowheads). In the case of PBMCs (Figure 2B), FLEX had slightly more features detected than 3pGEX, with HIVE and Evercode showing lower detection. Interestingly, HIVE exhibited a wider distribution of both features and total counts. Similar sample characteristics were observed at the cell-type level for both leukocytes (Figure S4A) and PBMCs (Figure S4B) including consistent distribution of cell types across sites and technologies, thus suggesting successful integration of datasets (Fig. S5 A and B). The exception is the presence of platelets and an “unknown” cluster likely containing debris or ambient RNA in the HIVE PBMC sample (Figure S5B), which likely account for the wider distribution of feature counts in that dataset.

To facilitate the comparison of cell-type proportions estimated by scRNA-seq and FACS, the detailed assignments were collapsed to generalized cell types (Figure 3B and D). Overall, cell-type proportions between the scRNA-seq and FACS were generally concordant. Approximately 50% of the cells in the leukocyte groups are granulocytes with most being neutrophils. As expected, this population is a much lower fraction in the PBMC samples. In the leukocyte data, there is close agreement between the HIVE 1-day and HIVE 4-week groups, indicating that this storage period does not impact cell-type proportions. 10x 3pGEX demonstrated the lowest proportion of granulocytes; they are still abundant. T- and NK-cell proportions were also enriched in 10x 3pGEX samples compared to all other single-cell technologies, and they more closely resembled the proportions of these cell types from FACS data. In contrast, FLEX and HIVE data demonstrated enriched proportions of monocytes relative to FACS and 10x 3pGEX. For the PBMC samples, we note strong agreement between the FACS data and both 10x technologies, while HIVE and Evercode technologies exhibited a reduction in the proportion of T-cells present and an increase in the proportion of monocytes. All single-cell technologies demonstrated a slight enrichment of B-cell proportions relative to FACS (Figure 4). Together, these results suggest single-cell preservation platforms achieve similar cell-type composition to each other and to FACS data; however, some exceptions exist for specific cell types.

**Figure:**
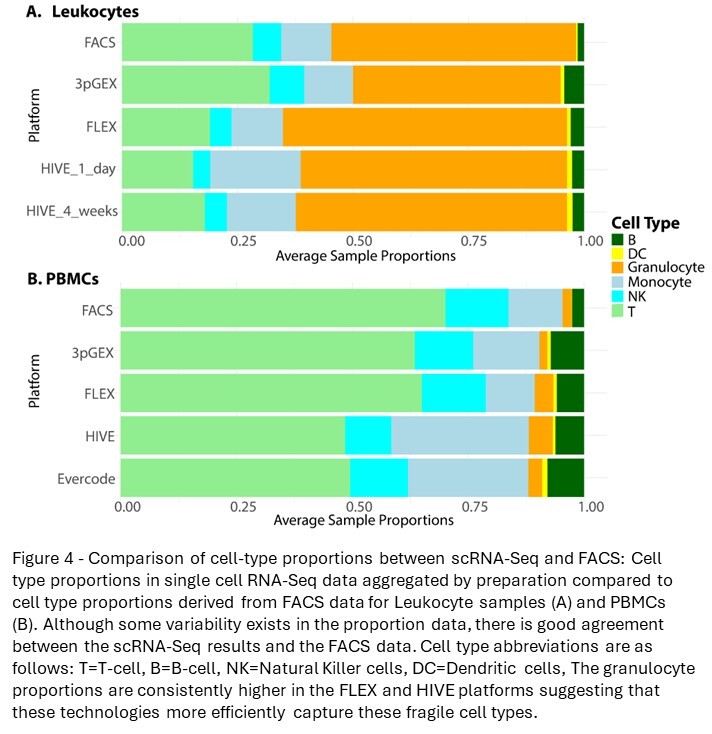


To assess the similarity of leukocyte expression profiles across different technologies, correlation analysis was performed on the averaged data calculated using the “Seurat” function AggregateExpression and the SCT assay. Importantly, genes not targeted by the 10x FLEX probeset were removed from this analysis. Correlation coefficients were high across all technical replicates (minimum of 0.93), sites (minimum of 0.91), and technologies (minimum of 0.91) with an overall minimum correlation coefficient of 0.75. Hierarchical clustering of correlation coefficients reveals strong clustering by technology, suggesting some level of platform-specific expression (Figure 5A). The HIVE and 10x data form two distinct clades, with the 1-day samples (10x 3pGEX) being distinct from the 28-day samples (FLEX). Within the two 28-day clades, the HIVE site samples and the A and B replicates intermix with one another without discernible metadata associations, which suggest that the site and technician variables have minimal contribution to the resulting expression data. However, within the 10x FLEX 28-day clade, site 1 and 4 samples are distinct from the site 2 and 3 samples, and the A and B replicates within the site 1 and 4 samples are clustered together. This site distinction in the 10x FLEX data aligns with the QC parameter profiles (Figure 3A) and may indicate that the 10x FLEX processing routine is more susceptible to technical variation from sample processing differences compared to the HIVE. Despite these differences, these data indicate each platform obtains leukocytes with highly similar expression profiles.

**Figure:**
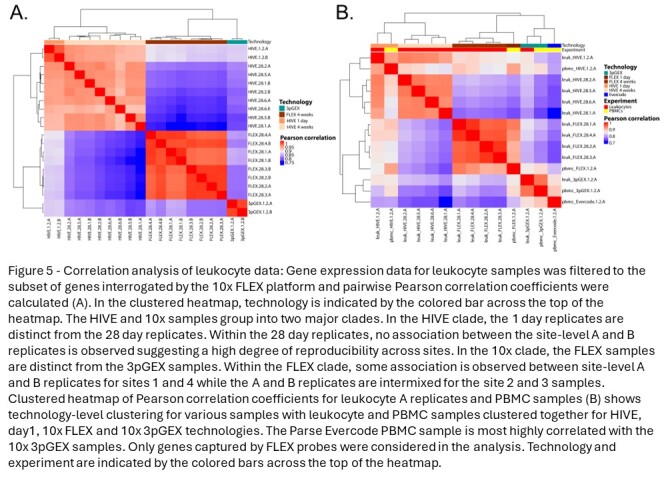


To investigate reproducibility of the fixation and storage process between replicates processed by different technicians at both the same site and across different sites, differential expression analyses between specific sample sets were performed (Figure 6A). The violin plot in the top panel shows log2 counts for each sample under consideration, and the box plots in the bottom panel report the distribution of log2 fold changes for each A vs B comparison. The values for all tested genes are displayed, and horizontal boxes at the 0 line indicate that more than 75% of the genes in the test have log fold changes near 0. This is observed for all comparisons except for a single site (FLEX.28.4 A vs B). In this comparison, a subset of genes has fold changes >0 indicated by the box above the 0 line and likely reflects differences in the counts highlighted by the violin plot in the top panel. Note that a small number of outliers were observed in each comparison. In total for all comparisons, there are 12 outlier results that have fold changes outside the threshold of 1. These results come from seven genes: HBB, IGHD, IGHG2, IGHG3, IGKC, IGLC3, and MT-CO1. It should be noted that reporting significant genes as defined by fold change and p-value thresholds does not work well for these analyses because most of the fold changes are small. Taken together, minimal differential expression between A and B replicates was observed indicating that all platforms are highly reproducible when processed in the same facility.

**Figure:**
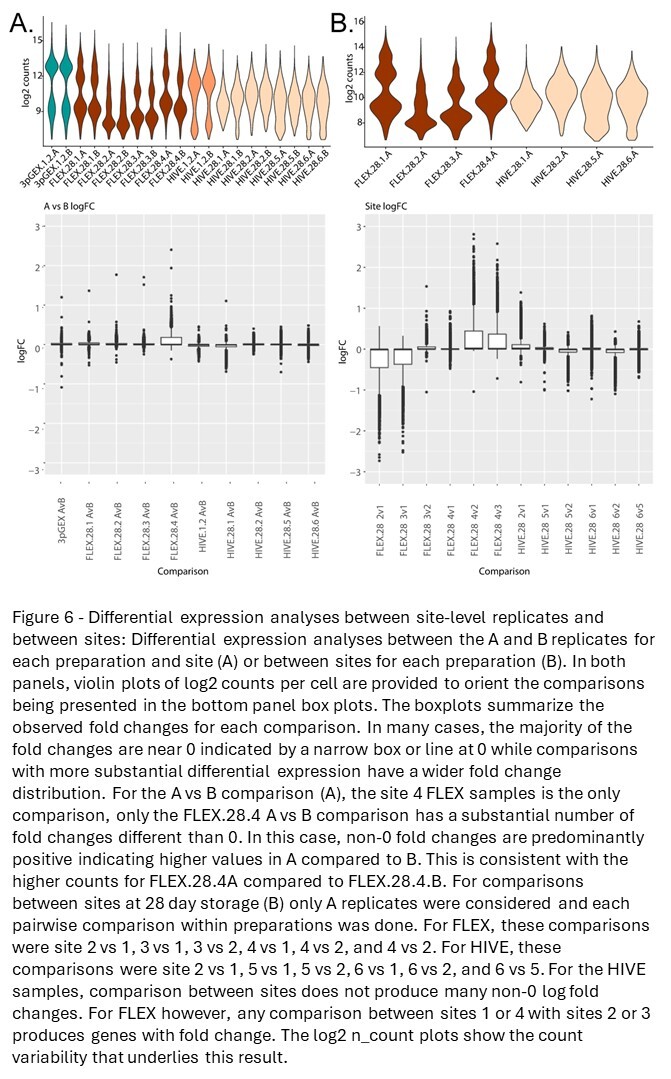


Differential expression analyses were also used to compare data generated at different sites (Figure 6B). Given that the previously noted correlation and differential expression data establish near equivalence between technical replicates, site comparisons were performed using only the A replicates. For completeness, site comparisons using the B replicates are provided in Figure S9, and the results are consistent with those observed for the A replicates, further supporting these conclusions. The top panel shows the log2 counts across sites, with log2 fold-change values from all pairwise comparisons represented as boxplots in the bottom panel. For the FLEX samples, we observe elevated log2 UMI counts for the site 1 and site 4 samples, compared with the counts from sites 2 and 3. This distinction can also be seen when comparing the number of differentially expressed genes between these groups with few DEGs detected when comparing sites 1 vs 4 and sites 2 vs 3, but a large number of DEGs comparing either site 1 or 4 with site 2 or 3. For the HIVE samples, none of the site comparisons produced substantial numbers of DEGs consistent with the previously presented correlation data.

To further characterize the library complexity of data generated across different platforms and sites and the differential expression result observed with site 4 in the 28-day FLEX samples, library complexity was modeled (Figure 7A). 10x FLEX BAM files were downsampled and re-counted using a custom PySAM script that accurately reproduces Cell Ranger UMI calling logic. The resulting reads and /UMI curve were fit using the Michaelis-Menten equation, where Vmax = max # of detectable UMIs and Km = reads per cell at 1/2 saturation. These Vmax values are plotted in the bar chart in Figure 7B and compared to the results for the 10x 3pGEX technology, filtering for genes included in the FLEX probe pool. Lower library complexity was observed in samples prepared at sites 2 and 3 compared to samples prepared at sites 1 and 4. This is supported by the barcode rank analysis, showing higher overall UMI counts in site 1 and 4 samples despite having grossly similar profiles (Figure 7C). Substantial variability was also noted between A and B replicates from site 4 (Figure 7D). Close examination of how FLEX samples were handled at each site revealed slight differences in the process that could contribute to the observed variation in UMI counts across sites. It was noted that sites 1 and 4 samples utilized fixed-angle rotors for centrifugation and appeared to have lower cell counts following probe hybridization, while sites 2 and 3 used swing-bucket rotors for these steps. These deviations in centrifugation do not appear to influence the proportion of neutrophils from these samples, which have lower RNA content and could account for this result nor does it explain the variability between the A and B replicates only at site 4. Together, these results indicate that the additional sample preparation steps required at each site for the FLEX assay can introduce variability, and care should be taken to standardize these procedures as much as possible.

**Figure:**
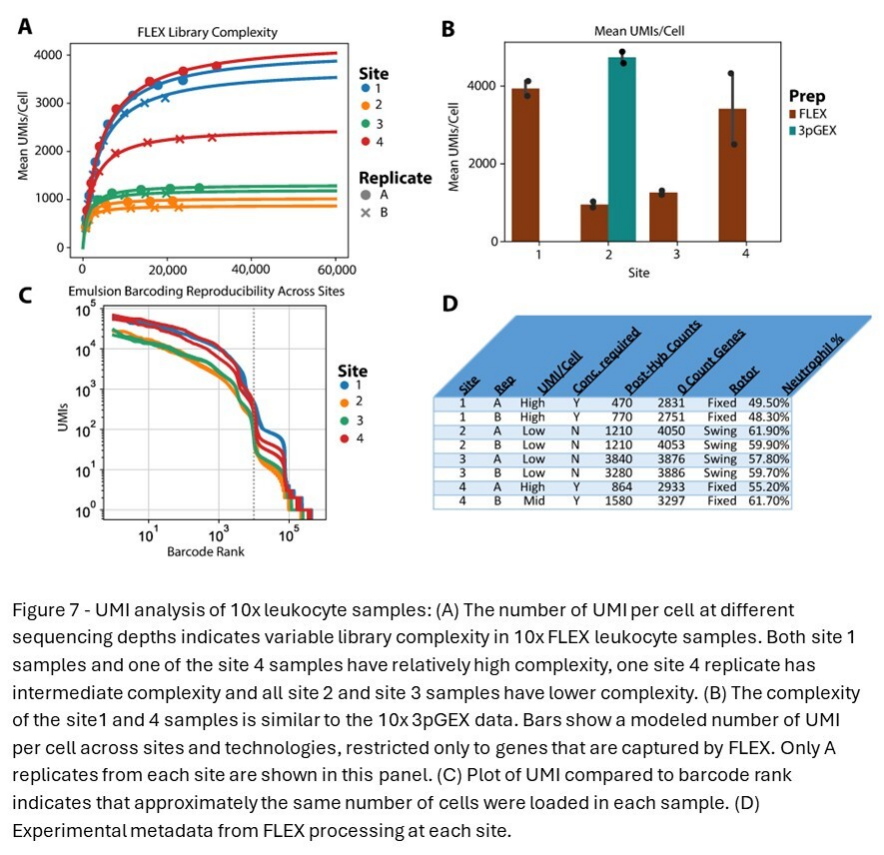


Due to the incompatibility of RBC-depleted total leukocytes with the Parse Evercode v2 chemistry, an additional human PBMC sample was collected to allow assessment of all three technologies. Correlation analysis was performed (Figure 5B). Samples were clustered according to technology and to a lesser degree by sample type (i.e., leukocyte vs PBMC). This can be seen in the distinct clustering of FLEX and HIVE samples, as well as 3pGEX and Evercode samples, though the latter two technologies are most similar to one another. Interestingly, while HIVE 1-day and 28-day samples exhibit strong correlation coefficients (Pearson >0.9), they cluster separately from one another suggesting differences in expression profiles based on the length of storage prior to processing. To investigate this further, differential expression was performed between leukocyte HIVE day-1 and day-28 samples (Figure S7), and no genes with a log fold change of >1 were observed. These observations suggest that while a high degree of consistency is observed, the choice of technology is not without impact, and subtle differences in results can be detected.

In order to provide insight into the correlation analyses, the molecular biology underlying each method was considered in more detail using PBMC alignment data, and the results are shown in the plots shown in Figure S8. The Evercode and HIVE alignments are roughly uniformly distributed along the transcript while, as expected, the 10x 3pGEX reads are concentrated at the 3’ end of the transcripts (Figure S8A). The 10x FLEX alignments are probe-based, and therefore average coverage distributions could not be calculated. Expression of a selected example gene (CD74, Figure S8B) shows the expected coverage distribution for reads originating from each platform (Figure S8C). This Integrated Genomics Viewer (IGV)^33,34^ image of the CD74 locus with the PBMC BAM files is loaded for display. 10x 3pGEX data appear at the 3’ end of the gene, while three FLEX probes (red arrowheads, FLEX probe track) can be seen overlapping three internal exons. The centrally located probe spans an exon/intron junction resulting in a split appearance. The whole-transcript nature of the HIVE and Evercode reads is apparent from the alignments where coverage is highest over exons across the gene body. Of note, nonexonic coverage is observed in the 10x 3pGEX, HIVE, and Evercode tracts. Examination of alignment in this region suggests that these reads may be caused by genomic regions that are rich in A or T bases (not shown) and is consistent with previous studies demonstrating internal priming of poly-A/T tracts in nuclear pre-mRNA transcripts from technologies using oligo-dT-based RT priming strategies.^35^

## Discussion

Single-cell RNA sequencing has transformed our understanding of biology but has historically been limited to the analysis of high-quality, fresh specimens processed at the site of collection. This requirement has hindered widespread adoption of this technique for clinical and other specimens where samples may be procured at sites without access to single-cell instrumentation or for samples that are collected over time and would benefit from additional data before committing to downstream processing. The availability of commercial solutions to preserve specimens prior to single-cell analysis through cryopreservation and/or fixation addresses many of these issues, but systematic assessments of these approaches are lacking. Herein, we examine the ability of the 10x Genomics FLEX, Honeycomb Bio HIVE, and Parse Evercode technologies to capture gene expression profiles from RBC-depleted total human leukocytes and PBMCs from a single individual and compare the relative cell-type proportions to 21-color flow cytometry data generated in parallel. While prior studies explored the differences between various scRNA-seq technologies by comparing metrics such as cost, performance, and sample compatibility,^16–19,36^ ours is the first to assess the reproducibility of these methods in terms of both the sample preservation process as well as the downstream capture and preparation of sequencing libraries across geographically separated core facilities.

In addition to the sample preparation challenges, components of downstream analyses were carried out in different core facilities. Because the deployment and management of consistent computing environments across sites can be difficult, we utilized a Docker image prepared by our team containing all necessary software packages. This image was run on various high-performance computer environments using singularity.^37,38^ This approach enabled work to progress in different sites using identical software, and it is ideal for core facilities that wish to promote collaboration and reproducibility.

Both the 10x FLEX and Honeycomb HIVE kit performed well in preserving human leukocytes specimens, including retention of granulocyte populations with relative proportions of cell types comparable to the “gold standard” flow cytometry dataset. While three of the four sites were able to generate sequencing libraries from total human leukocytes using the Parse technology, very few cells were captured, and the data quality was poor (data not shown). That the PBMC samples were successful suggests that granulocytes may be problematic for this method. The findings from FLEX and HIVE are consistent with observations from Hatje et al.,^36^ and while they were able to generate libraries containing granulocytes, those populations are significantly underrepresented in their dataset. In contrast to Hatje et. al.,^36^ we observe granulocytes in the 3pGEX dataset; however, they are underrepresented compared to the other technologies. This result is likely due to the optimized sample collection procedure used for this study where cells were captured or preserved within 2 hours of the initial blood draw. Overall, the detection of granulocytes is an advantage of the FLEX and HIVE technologies compared with Parse or the processing of fresh samples using 3pGEX. PBMCs were also examined using 10x 3pGEX, 10x FLEX, Honeycomb HIVE, and Parse Evercode. All technologies produce high quality gene expression data that can be used to identify the expected cell types in proportions that are comparable to each other and concordant with flow cytometry measurements.

The ability to detect granulocytes has long been a challenge for single-cell studies and even with their improved retention using preservation-based methods, computational solutions are needed to distinguish granulocyte-associated barcodes from those harboring ambient RNA or platelets, all of which have similar RNA content. Herein, we considered standard QC parameters combined with analysis of cluster-specific markers to distinguish cell-associated droplets from acellular debris. This approach is effective at identifying cell-containing droplets and can be used to improve the recovery of RNA poor cell types independent of the platform used. Despite small exceptions, the cell-type proportions we observe are similar in data from different platforms. This is consistent with previous reports that the 10x 3pGEX platform can capture granulocytes using specific sample preparation procedures and a modified analysis routine to include low RNA content cells.^36,39^

Despite the high-level agreement between methods, detailed comparisons at the gene level reveal significant differences across technologies. Within the leukocyte dataset, correlation analysis between samples from 3pGEX, FLEX, and HIVE demonstrate clustering by technology with the stronger cross-site correlations observed for the 10x samples compared with HIVE. When including samples from the PBMC dataset, we again see strong clustering based on the technology used rather than the sample type (i.e., leukocyte or PBMC). One possible explanation is the considerable difference in assay design across the technologies tested. We explored the distribution of read alignments and found that, as expected, reads were concentrated in the 3’ region for samples prepared using 10x 3pGEX while the HIVE and Parse kits exhibit more uniform, full-length transcript coverage (Figure S8). We also observed similar levels of mitochondrial abundance for both leukocytes and PBMCs except for 10x FLEX, which excludes many mitochondrial genes from the probe set (Figure 2B). Despite these differences, broad concordance in cell-type abundance, sample quality, and total gene detection is consistent across technologies, thus enabling the successful integration of the datasets and comparison of QC metrics including gene and UMI counts per cell, which were similar across all technologies.

Our unique study design also allowed us to assess variation at the level of sample preservation, as well as across sites using the same technology. Except for the site 4 FLEX replicates, we did not observe substantial differences in samples preserved by different technicians and analyzed them at the same performance site (A vs B replicates, Figure 5A). We do note variability between samples analyzed by the same technology across sites. Specifically, 10x FLEX samples from sites 1 and 4 show higher UMI counts per cell than those processed at sites 2 and 3. While the precise reason for these differences is not known, we note that sites 2 and 3 used swing-bucket rotors for sample centrifugation, as opposed to fixed-angle rotors at sites 1 and 4, with the latter resulting in lower cell counts following probe hybridization. We hypothesized this cell loss may have resulted in a reduction in the neutrophil/granulocyte fraction and the capture of more high RNA content cells. However, no significant differences in neutrophil abundance were observed for these samples; however, other populations may be affected (data not shown). This finding highlights the need to standardize downstream processing steps particularly for the FLEX method, where sample preservation is performed separately from single-cell capture, which can introduce additional variability into the workflow.

The study presented here does not attempt to be a comprehensive quantitative assessment of the impact of preservation on scRNA-seq data. Instead, we aimed to report on the experiences of multiple core facilities working with these technologies and to identify features of these workflows to be considered when deciding which to adopt for a given project. We find that strong correlations generally exist between replicate samples processed at different sites using the same method, but that differences in expressions exist between platforms. As a result, the chosen method should be based on the ability to accommodate the sample type and number of cells of interest as well as logistics of sample collection and downstream processing. With PBMCs as input, all platforms produce high quality data as assessed by the ability to identify cell types in proportions consistent with the other technologies. For more challenging cell types (such as total leukocytes), the 10x 3pGEX, 10x FLEX, and HIVE platforms performed well based on the metric of cell-type proportions. It is possible that if users wish to minimize technical variability and all library preparations will be done at one location, 10x FLEX might be the best choice. If data will be produced at a variety of locations, Honeycomb HIVE may be the best option. It is also possible that the length of storage could contribute to variation in the data as observed in the HIVE 1-day vs 28-day samples. This finding warrants further investigation across more timepoints and technologies to see if this effect is significant. While a detailed cost-benefit analysis is beyond the scope of this study, practical workflow considerations merit brief discussion. The 10x FLEX workflow requires fixation reagents and an additional on-site probe hybridization step but benefits from high multiplexing capacity and widely available support infrastructure. The Honeycomb HIVE system requires minimal hands-on time at the collection site (cells are simply loaded into the HIVE device and frozen), which makes it particularly well-suited for remote or low-resource collection sites; library preparation at the processing site is more labor-intensive and requires a specialized vacuum manifold apparatus. The Parse Evercode workflow offers highly scalable multiplexing with moderate reagent costs, but as noted in this study, this may not be compatible with all sample types and is a 2–3-day hands-on protocol that may be hard to implement in some facilities or as a core service. Overall, platform selection should weigh not only data quality but also reagent costs, hands-on labor, scalability to the number of samples, and the instrumentation available at both collection and processing sites. Several limitations of this study should be acknowledged. The PBMC follow-on experiment was performed at a single site after only 1 day of storage in contrast to the leukocyte samples, which were processed at four sites after 28 days. This asymmetry limits direct comparison of multisite reproducibility and long-term storage effects between the leukocyte and PBMC phases of the study. Future work should include PBMC samples processed at multiple sites and at extended storage time points to enable a more rigorous parallel comparison.

### Financial Support/Conflict of Interest

The authors have no financial support or associations that pose a conflict of interest.

CAW was partially supported by Cancer Center Support (core) Grant P30-CA14051 from the National Cancer Institute (NCI) to the Barbara K. Ostrom (1978) Bioinformatics and Computing Core Facility of the Swanson Biotechnology Center. MLM and SWP were supported by Delaware INBRE (NIH P20GM103446), Delaware CTR ACCEL (NIH U54GM104941), Delaware Biotechnology Institute, and the state of Delaware. FWK and OMW were supported by Cancer Center Support Grant (5P30CA023108) and NIGMS COBRE (P20GM130454) awards. The WashU GTAC@MGI is partially supported by NCI Cancer Center Support Grant P30CA91842 to the Siteman Cancer Center.

ABRF member cores that contributed to this work include: GSAF, University of Texas: RRID: SCR_021713; University of Delaware Bioinformatics Data Science Core Facility: RRID: SCR_017696; Dartmouth Genomics Shared Resource: RRID: SCR_021293; Center for Functional Genomics, UAlbany, Albany, NY: RRID: SCR_018262; Dartmouth Genomic Data Science Core: RRID: SCR_025383; Washington University School of Medicine Genome Technology Access Center Core Facility: RRID: SCR_001030; University of Wisconsin – Madison Biotechnology Center DNA Sequencing Facility: RRID: SCR_017759; and ICBR Gene Expression & Genotyping at University of Florida: RRID: SCR_019145.

## Supplementary Material

Table_S1

Figure_S1

Figure_S2

Figure_S3

Figure_S4

Figure_S5

Figure_S6

Figure_S7

Figure_S8

Figure_S9

## Data Availability

All raw and processed data associated with the study is available on GEO under Project GSE329418. The complete R object used for this study is available on Zenodo, under 10.5281/zenodo.19049577.
