# Supplementary figures and images for: Multisite Assessment of Methods for Cell Preservation Upstream of Single-Cell RNA Sequencing
Source: J Biomol Tech. 2026 Jun 8;37(2):9–27. doi: 10.7171/001c.162768 (PMC13252917; doi:10.7171/001c.162768)

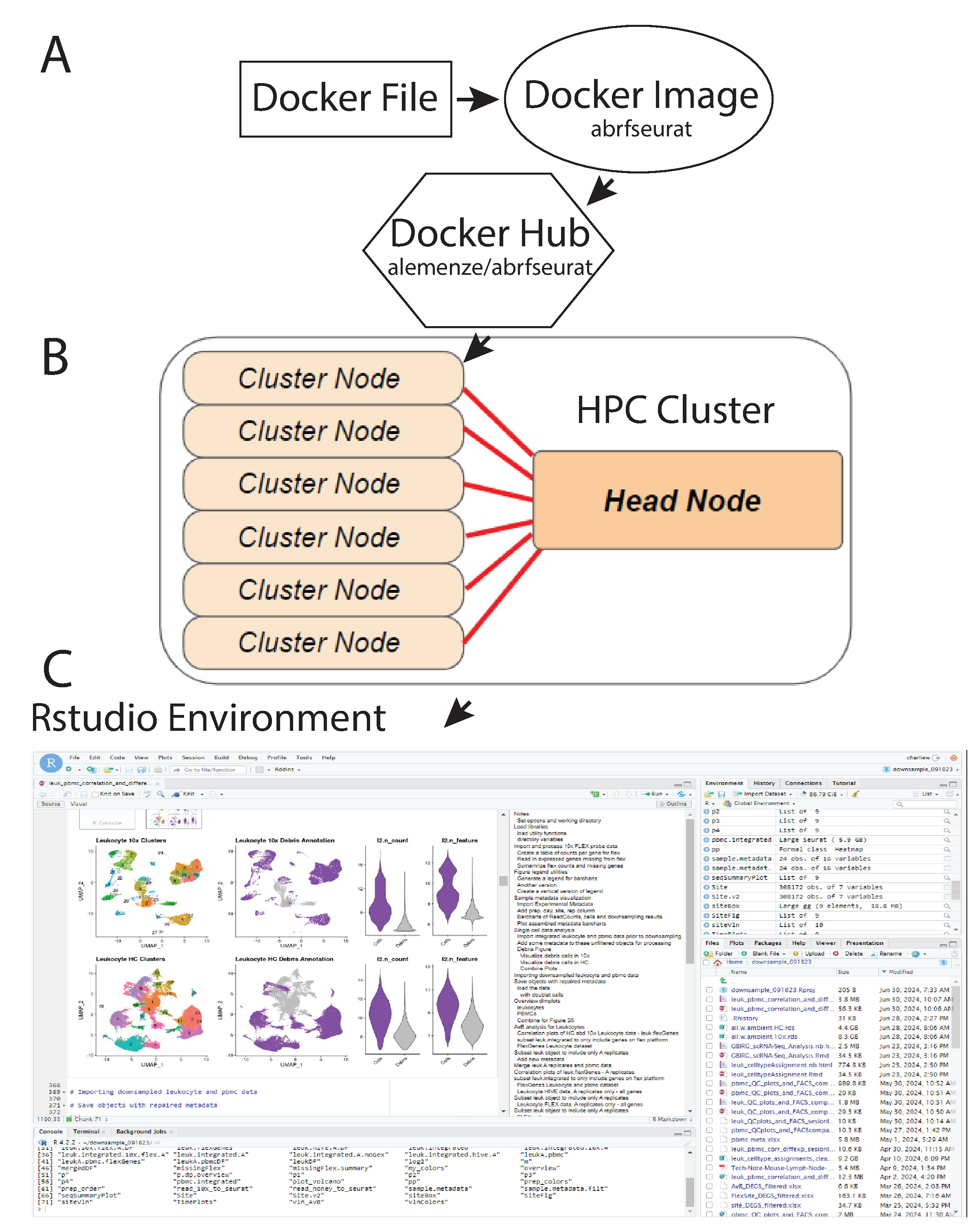

Supplement: Figure_S1 [file jbt_2026_37_2_162768_347240.png]

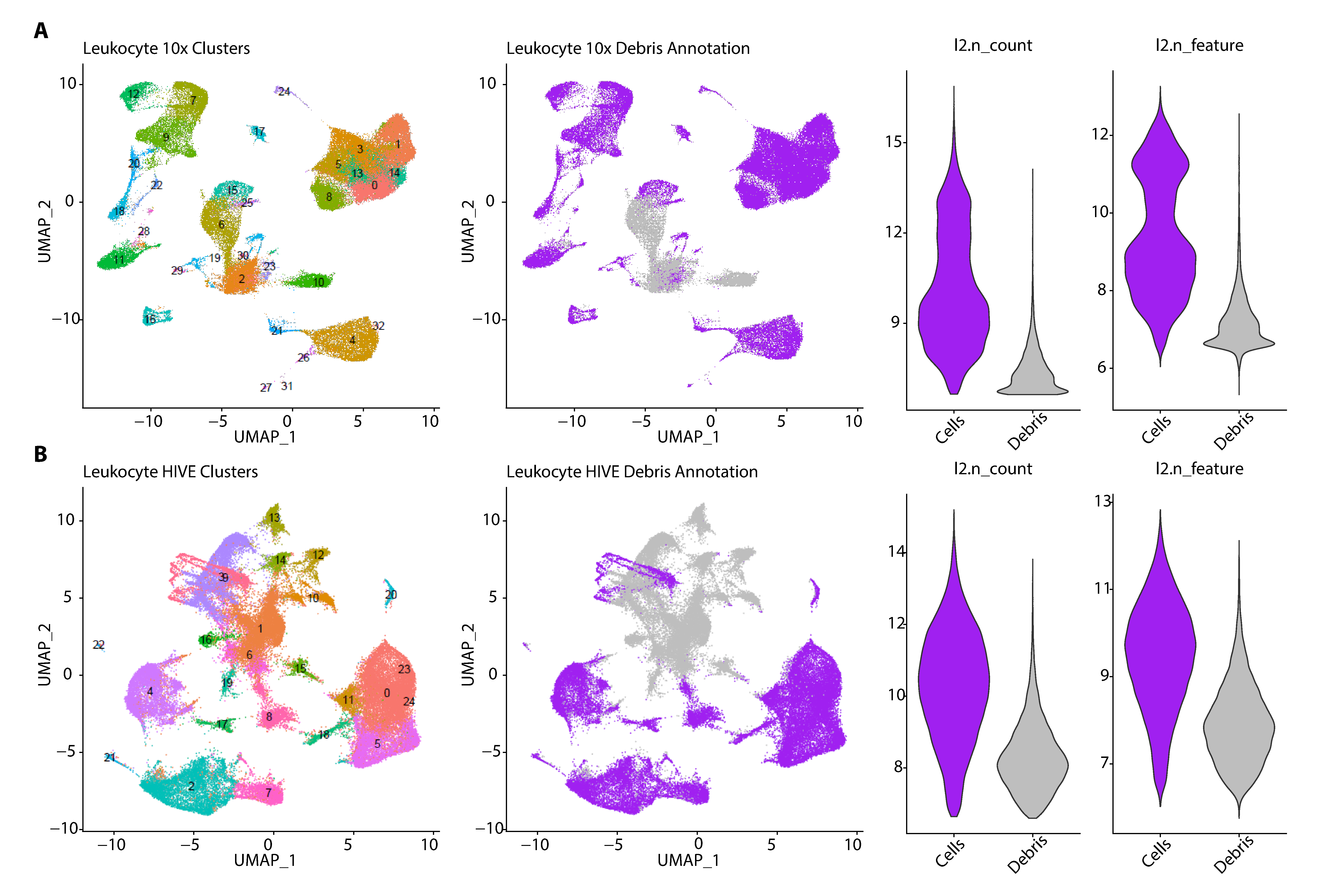

Supplement: Figure_S2 [file jbt_2026_37_2_162768_347234.png]

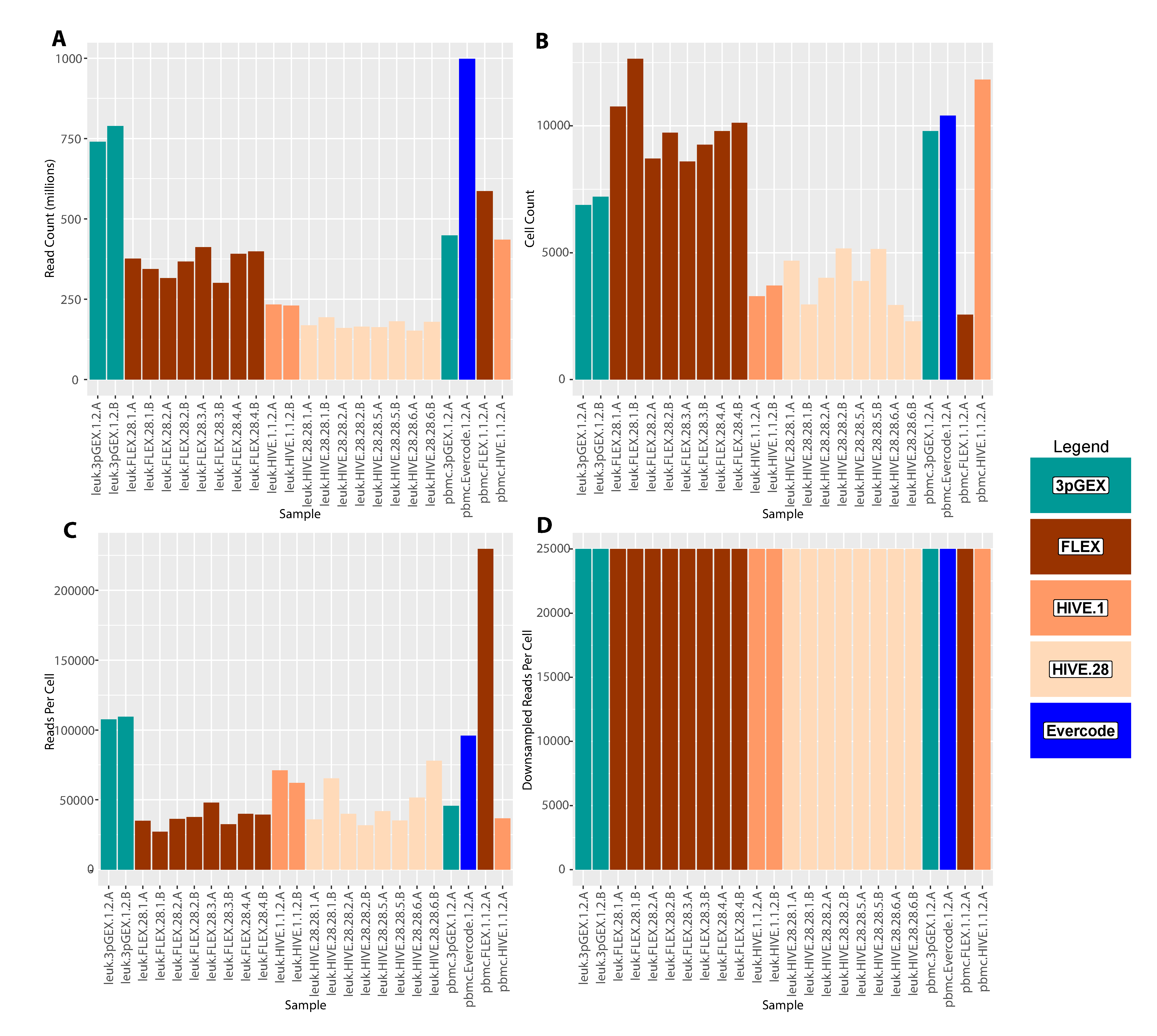

Supplement: Figure_S3 [file jbt_2026_37_2_162768_347235.png]

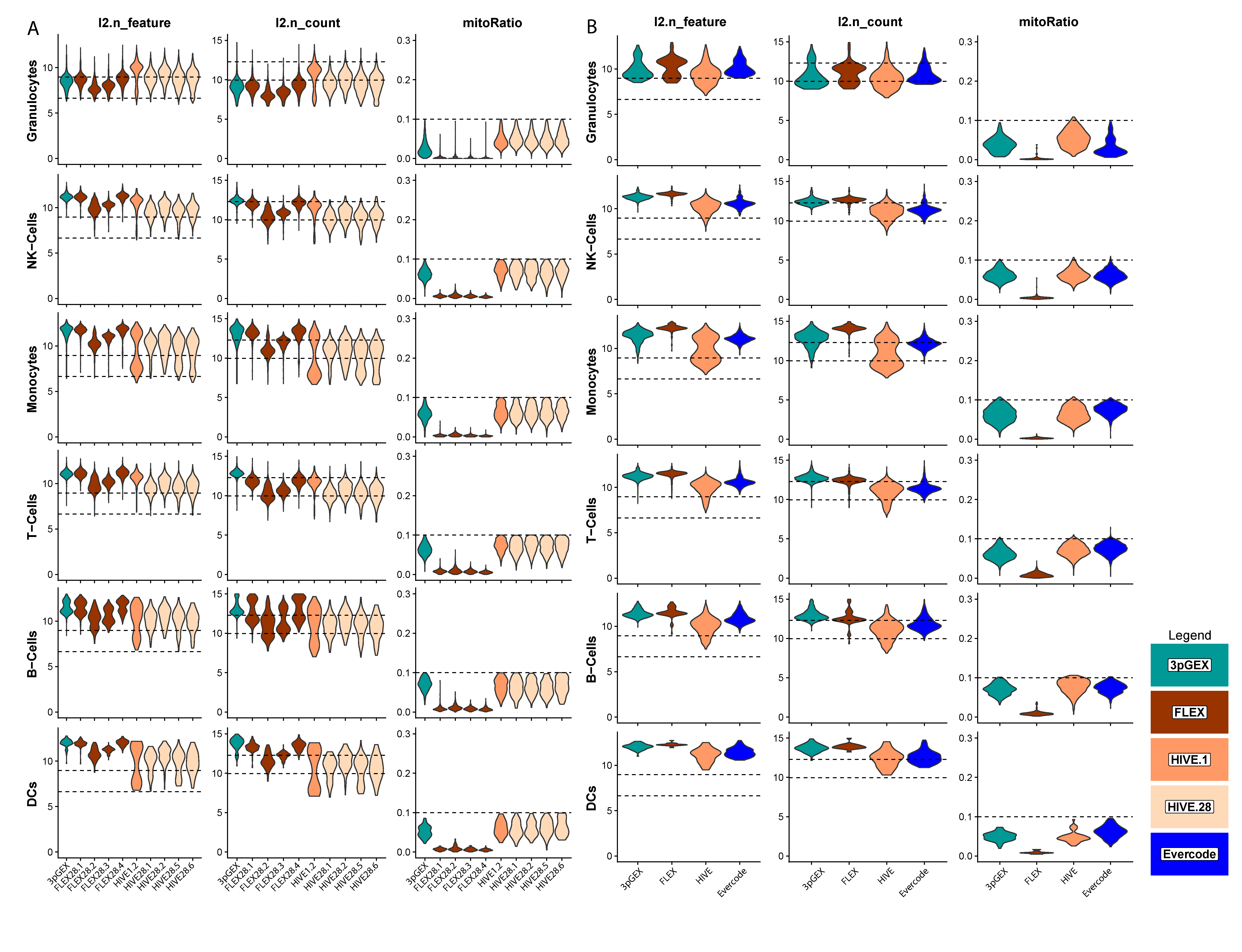

Supplement: Figure_S4 [file jbt_2026_37_2_162768_347230.png]

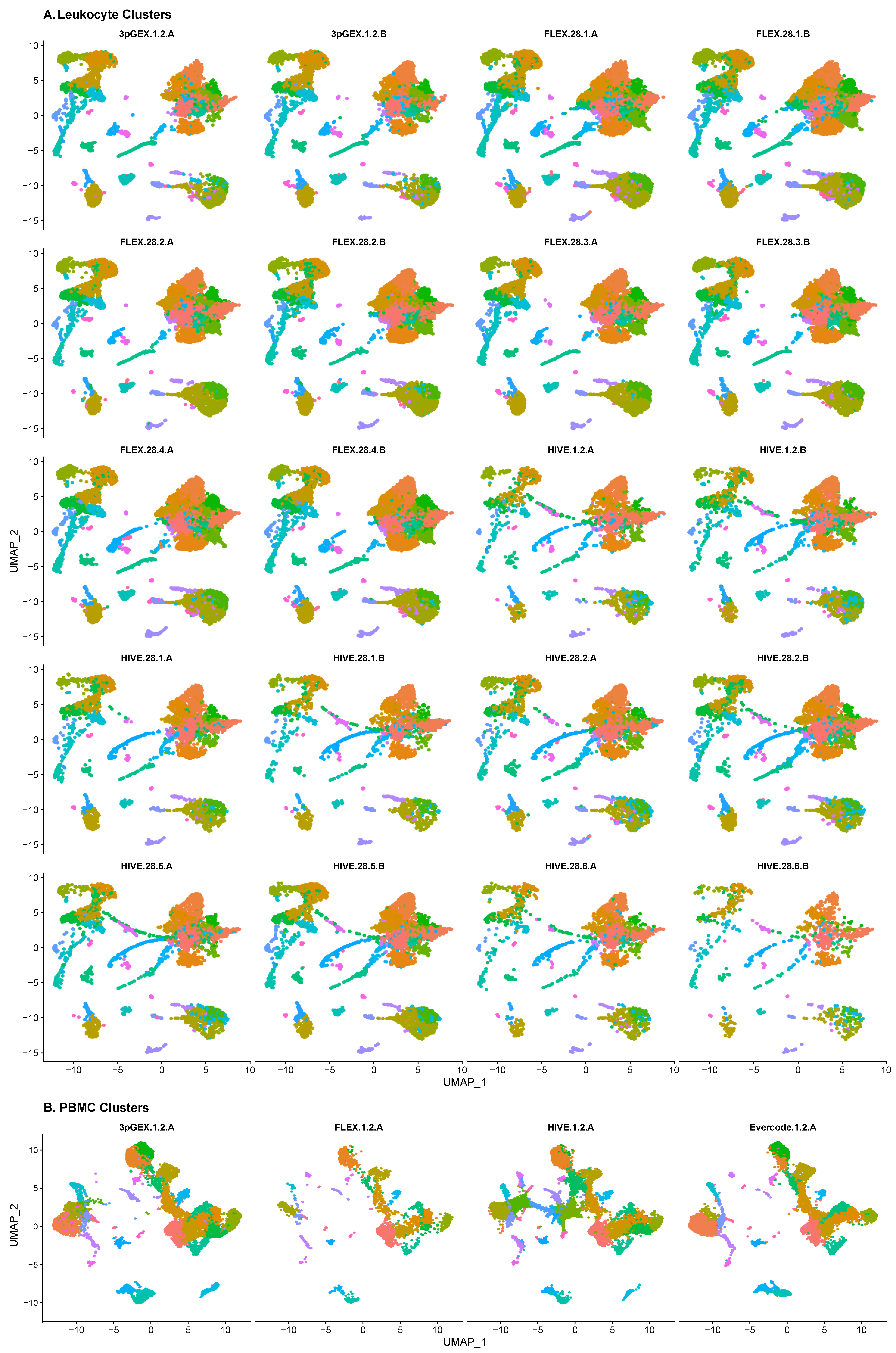

Supplement: Figure_S5 [file jbt_2026_37_2_162768_347227.png]

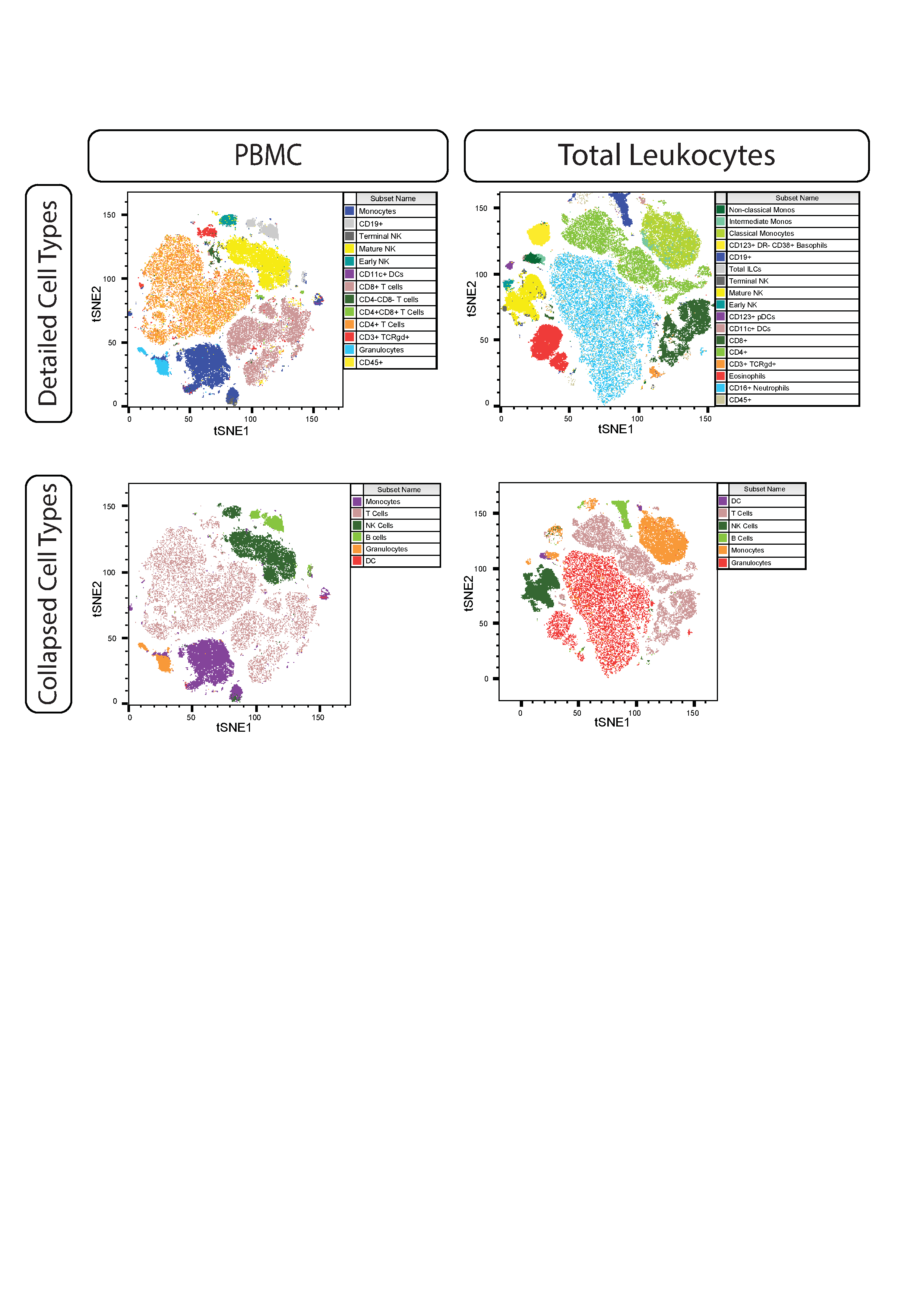

Supplement: Figure_S6 [file jbt_2026_37_2_162768_347239.png]

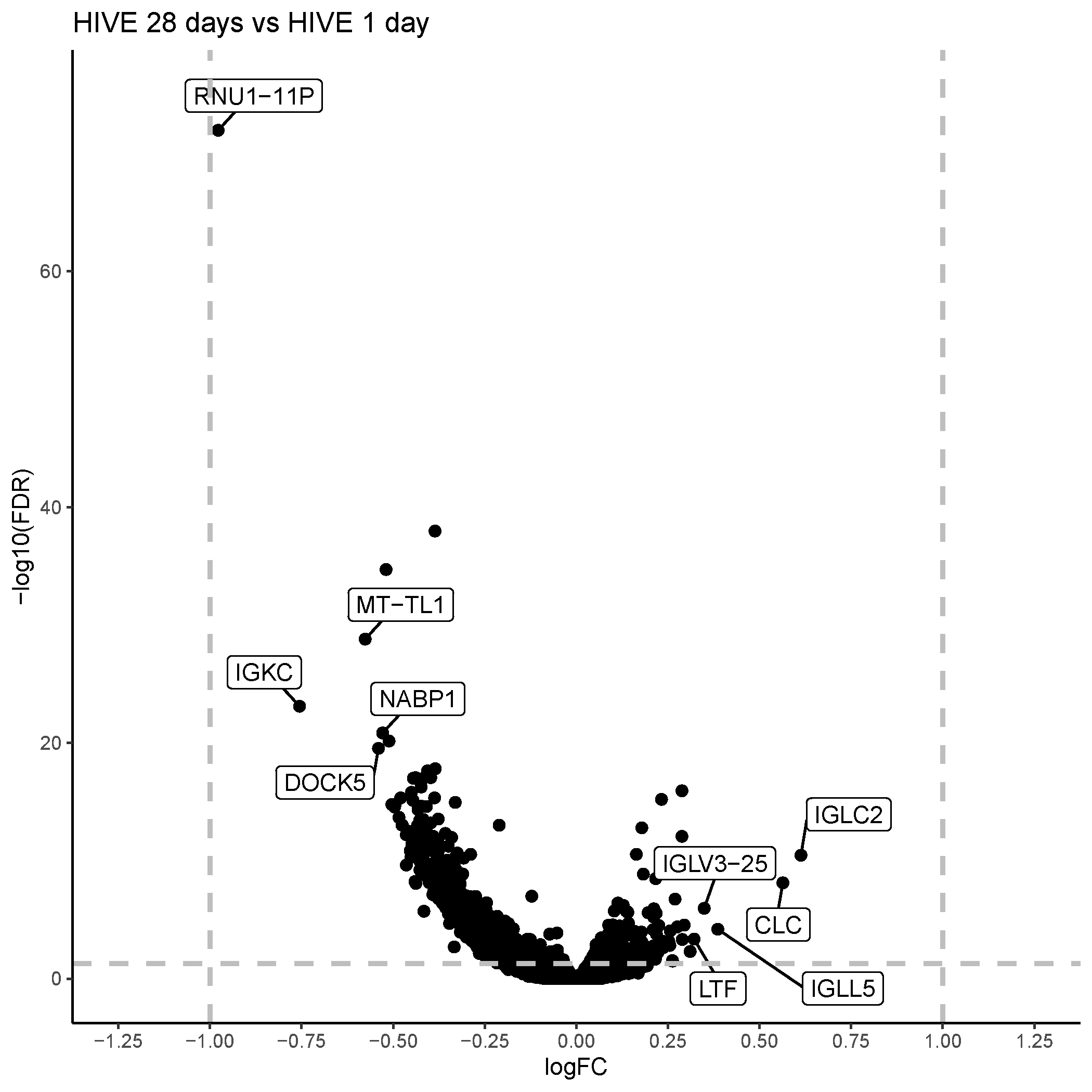

Supplement: Figure_S7 [file jbt_2026_37_2_162768_347237.png]

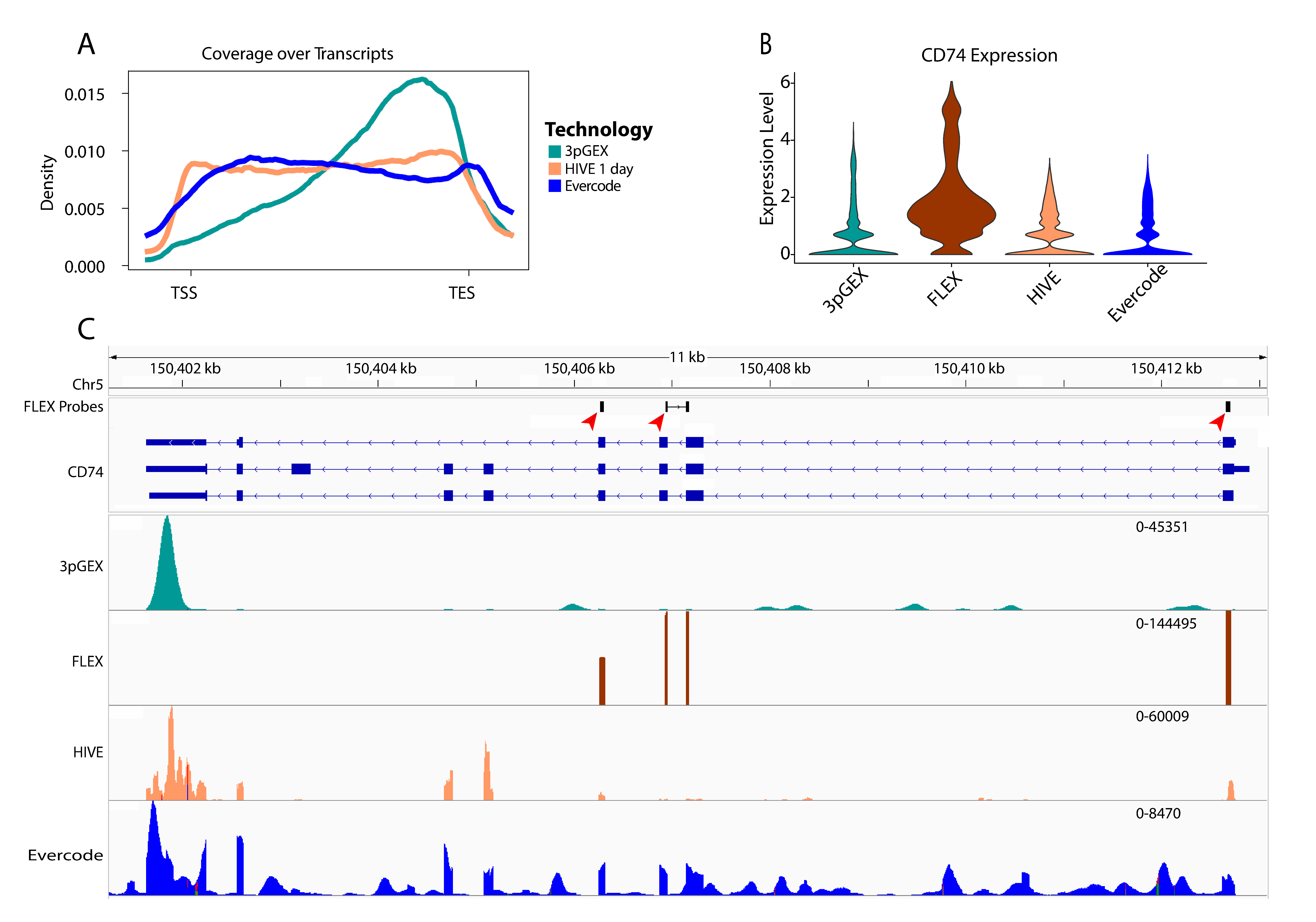

Supplement: Figure_S8 [file jbt_2026_37_2_162768_347228.png]

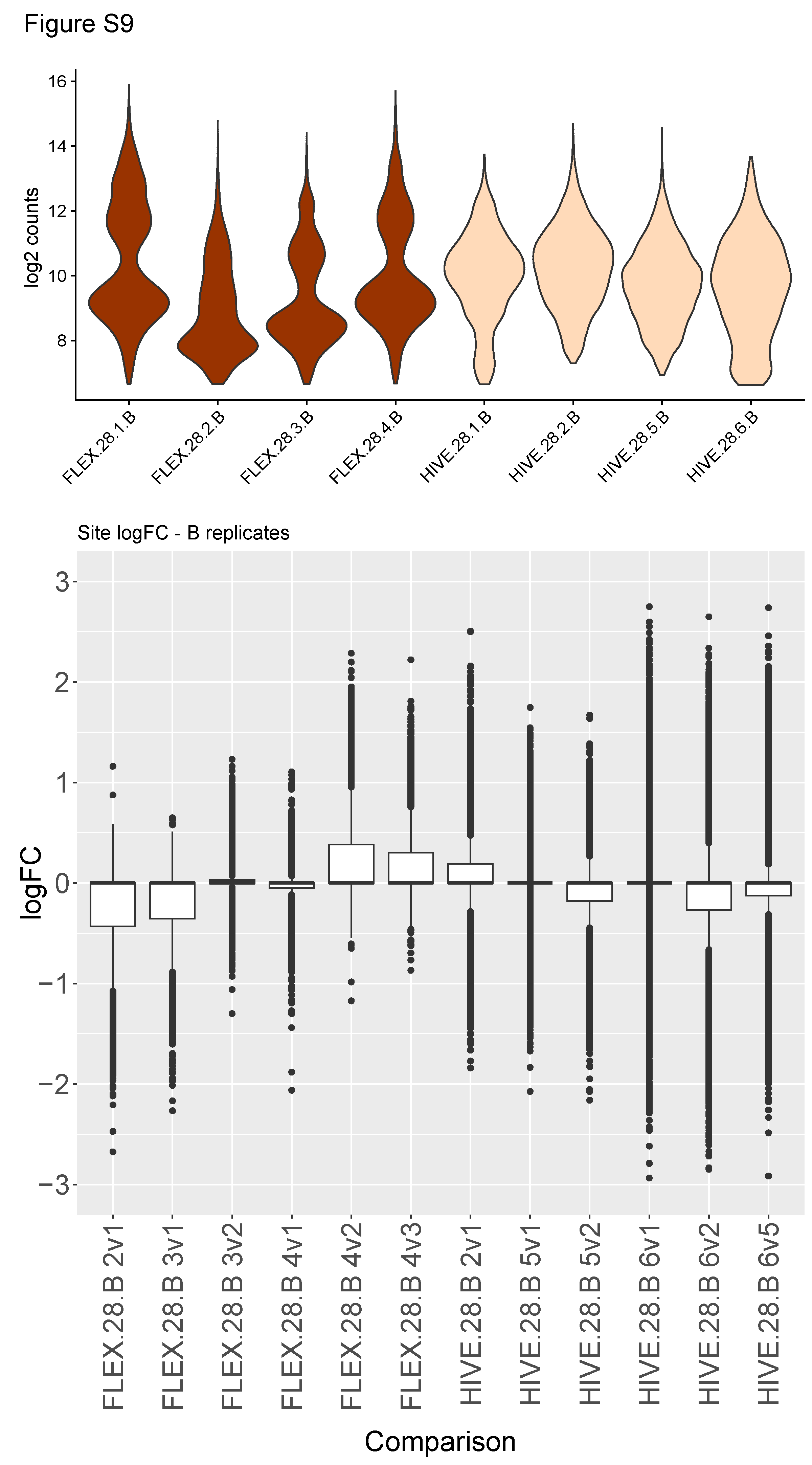

Supplement: Figure_S9 [file jbt_2026_37_2_162768_347236.png]
